# Extraocular Muscle Biopsy: Surgical Technique and Indications

**DOI:** 10.7759/cureus.29574

**Published:** 2022-09-25

**Authors:** Amine Razzak, Mohamed Bouazza, Houda Safwate

**Affiliations:** 1 Department of Ophthalmology, Faculty of Medicine, Mohammed VI University of Health Sciences (UM6SS), Casablanca, MAR

**Keywords:** orbital tumors, enlarged extraocular muscles, surgical technique, biopsy, extraocular muscle

## Abstract

Extraocular muscle biopsy is a technique used for the histological diagnosis of several orbital diseases; it could bring valuable assistance to the diagnostic process. The purpose of this article is to review the indications, surgical technique, and complications of this biopsy.

Extraocular muscle biopsy is frequently used in tumoral pathology, however, in inflammatory diseases, it is only used when facing a poor reaction to the treatment or in case of chronicity. The surgical procedure must be precise and least traumatic, in order to avoid intra- and post-operative complications, but also to preserve the integrity and function of the muscle. This procedure remains non-standardized and not devoid of complications, hence the need for a careful evaluation of the benefit/risk ratio before considering it.

## Introduction

Extraocular muscle biopsy is used in the diagnosis of several conditions such as congenital muscular dystrophies, inflammatory myopathies, and metabolic disorders [1].

This procedure is used for the histological diagnosis of several orbital diseases. Often used as a last resort, it can provide valuable guidance when encountering diagnostical difficulties, as well as histological evidence for an unfamiliar diagnosis. Even with the lack of documentation, the procedure is relatively easy and quick. However, it might come with some complications [2,3].

The purpose of this article is to review the indications, the surgical technique, and the complications of this biopsy.

## Technical report

Extraocular muscle biopsy can be performed under local, regional, or general anesthesia. For the biopsy, the extraocular muscles may be approached by an anterior transconjunctival or posterior orbital approach, depending on the location of the mass lesion. When the abnormal muscle is beyond reach (>18mm from the limbus), an orbital approach to muscle biopsy can be considered in correlation with the radiological location.

In the anterior transconjunctival technique, the tendon of the involved extraocular muscle is approached and isolated using either a fornix or limbus approach.

**Figure 1:**
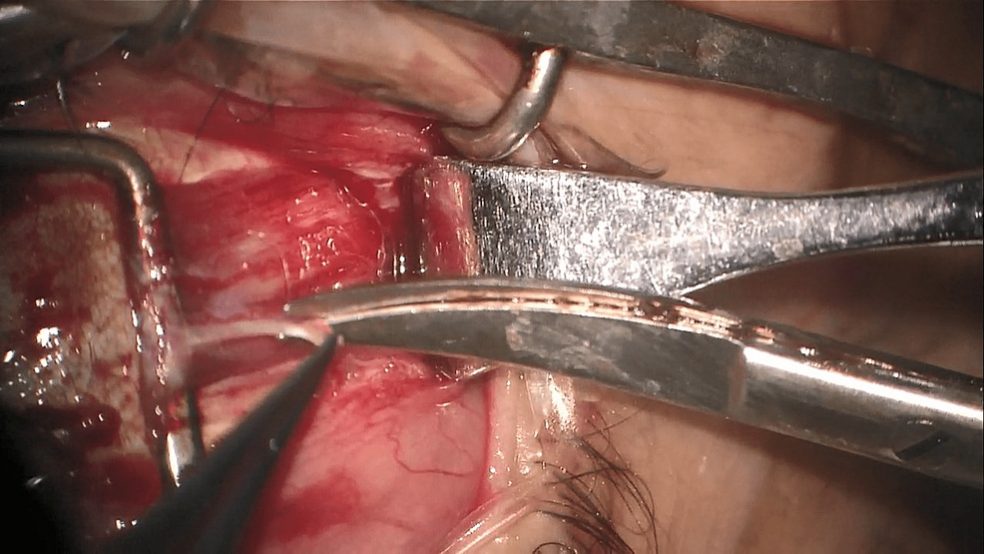
Biopsy of the superficial muscle fibers using scissors

**Figure 2:**
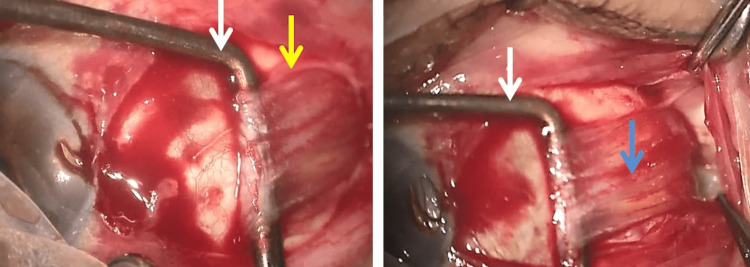
Capture of the lateral rectus muscle using a strabismus hook White arrow - strabismus hook; yellow arrow - muscle capsule; blue arrow - muscle belly cleared from muscle capsule.

The muscle belly is cleared from the muscle capsule using dissection scissors, afterwards, a strabismus hook is inserted between the muscle belly and the eye globe, and then a second hook is placed posteriorly to the first (Figure 1). The belly muscle is suspended, divided into longitudinal muscle fibers then retrieved by scissors (Figure 2).

If the defect in the biopsied muscle is too important, it can be closed by a ligature of 6-0 or 7-0 absorbable suture. Finally, the conjunctiva is closed in standard fashion using 7-0 or 8-0 absorbable suture. Steroids, antibiotics, and painkillers are prescribed right after the surgery. The ideal biopsy yields sufficient tissue for analysis without compromising the function of the tissue and muscle sampled.

**Figure 3:**
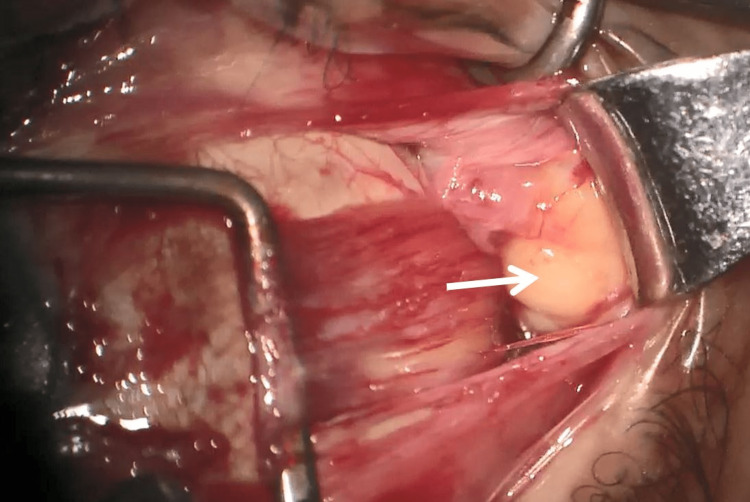
Discovery of a mass near the lateral rectus muscle

**Figure 4:**
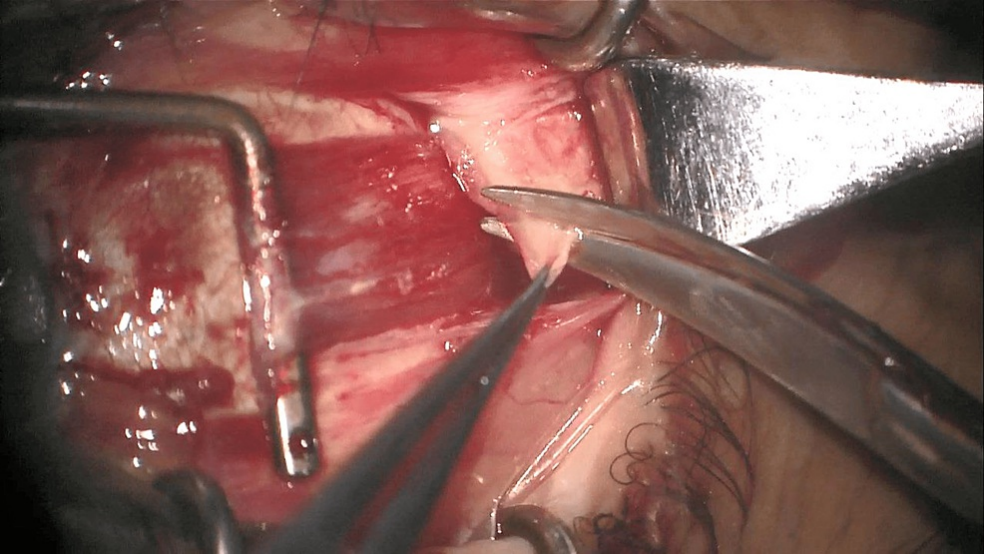
Biopsy of the mass near the lateral rectus muscle

In order to avoid mechanical complications, the biopsy must be done parallel to the muscle fibers and must concern the superficial fibers at the level of the orbital surface of the rectus muscles; other macroscopically abnormal tissues must also be taken [4] (Figures 3-4). A new sutureless technique of extraocular muscle biopsy was described using muscle hooks and a malleable retractor [5].

## Discussion

The procedure is designed to provide an adequate sample of muscle for analysis while avoiding post-operative complications. When facing hypertrophy of the extraocular muscles, the used approach remains controversial [4].

According to several authors, the main indication of extraocular muscle biopsy is when suspecting tumoral growth near or within a muscle. This procedure is also recommended in cases of chronic inflammation or atypical muscle invasion (especially if the MRI shows structural anomalies without clear etiology) [2,5,6]. However, the biopsy is not an option for patients with an infected orbital area (orbital cellulitis, for instance).

The biopsy must concern the pathological extraocular muscles, however, when several muscles are affected, the benefit/risk ratio must be assessed, in fact, the surgical complications related to the biopsy of the superior and lateral rectus are more frequent than that of the medial and the inferior rectus muscles, and the inferior oblique [6]. The procedure remains an invasive one that can cause several intra and post-operative complications [3,6].

In the following table, we have listed the most frequent complications seen during or after the procedure (Table 1).

**Table 1:** Intra and post-operative complications of extraocular muscle biopsy

Intra-operative complications	Post-operative complications
Tear and retraction of muscle and tendon, Hemorrhage	Postoperative pain, Iatrogenic diplopia, Adhesion and muscle fibrosis, Upper lid ptosis, Infections (myositis, orbital cellulitis)

## Conclusions

The etiological diagnosis of extraocular muscle enlargement requires a methodical and rigorous diagnostic approach and a biopsy can be helpful in this regard. However, this invasive procedure should only be proposed in cases of infiltrative, chronic, or atypical inflammatory eye disease. Poorly indicated, this technique has not thoroughly been described in the literature despite being a simple and quick procedure that can give valuable information. This procedure remains non-standardized and not devoid of complications, hence the need for a careful evaluation of the benefit/risk ratio before considering the biopsy.
